# The fungus *Aspergillus niger* consumes sugars in a sequential manner that is not mediated by the carbon catabolite repressor CreA

**DOI:** 10.1038/s41598-018-25152-x

**Published:** 2018-04-27

**Authors:** Miia R. Mäkelä, María Victoria Aguilar-Pontes, Diana van Rossen-Uffink, Mao Peng, Ronald P. de Vries

**Affiliations:** 1 Fungal Physiology, Westerdijk Fungal Biodiversity Institute & Fungal Molecular Physiology, Utrecht University, Utrecht, The Netherlands; 2 Department of Microbiology, Faculty of Agriculture and Forestry, University of Helsinki, Helsinki, Finland

## Abstract

In nature, the fungus *Aspergillus niger* degrades plant biomass polysaccharides to monomeric sugars, transports them into its cells, and uses catabolic pathways to convert them into biochemical building blocks and energy. We show that when grown in liquid cultures, *A*. *niger* takes up plant-biomass derived sugars in a largely sequential manner. Interestingly, this sequential uptake was not mediated by the fungal general carbon catabolite repressor protein CreA. Furthermore, transcriptome analysis strongly indicated that the preferential use of the monomeric sugars is arranged at the level of transport, but it is not reflected in transcriptional regulation of sugar catabolism. Therefore, the results indicate that the regulation of sugar transport and catabolism are separate processes in *A*. *niger*.

## Introduction

Plant biomass is a complex entity containing both structural and storage polymers that consist of several sugar monomers. *Aspergillus niger* is a ubiquitous filamentous ascomycete fungus, which efficiently degrades all plant polysaccharides through a wide range of extracellular carbohydrate acting enzymes (CAZymes)^1^. The resulting sugars are taken up by the fungal cell and converted to energy or biomolecule precursors through a variety of metabolic pathways^2^. *A*. *niger* is also one of the main industrial producers of enzymes for the conversion of plant-based feedstocks to fermentable sugars^3^. Although nowadays more complex substrates are used as a carbon and energy source in fungal fermentations, growth on monomeric sugars is utmost important to understand the physiology of the fungus. A fine-tuned regulatory mechanism enables *A*. *niger* to utilize the available carbon source in the physiologically most beneficial manner.

During growth in natural biotopes, fungi are confronted with a heterogeneous mixture of carbon sources of which, due to substrate consumption that supports fungal growth, the composition changes over time. For fungi to thrive and propagate, it is essential that the set of expressed genes enables utilization of the energetically most optimal carbon source, and adapts in response to changes in the substrate composition. The expression of the genes required for the extracellular degradation of the polysaccharides and the subsequent intracellular metabolic conversion of the sugar residues is therefore controlled by a set of transcriptional activators and repressors that respond to specific inducers^4^. Carbon catabolite repression, mediated by CreA, ensures that the presence of a preferred carbon source (e.g. glucose) prevents expression of genes involved in utilization of less-preferred carbon sources^5^. CreA has been hypothesized to be the key mechanism in fungi to ensure the optimal match to available substrate, as increasing free sugar concentrations in fungal habitats increase repression of genes encoding polysaccharide-degrading enzymes^4^. To achieve carbon repression through CreA, high transcript levels of *creA* are required, as well as glucose transport and at least partially the presence of CreB^6^. CreB is part of the CreC-CreB deubiquitination complex that is essential for CreA function and stability^7^. Deletion of CreA can result in upregulation of many genes, but also in a more clear distinction between the sugars that induce expression of different sets of plant biomass degradation related genes, such as has been shown for a set of pectinolytic genes in *A*. *niger*^8^.

However, CreA is not the only mechanism for carbon repression. It has been shown that two α-rhamnosidase encoding genes of *Aspergillus nidulans* were repressed in the presence of several carbon sources without the involvement of CreA^9^. In addition, low carbon source conditions resulted in upregulation of several amylolytic genes in *A*. *nidulans* in both the wild type and a *creA* mutant, suggesting repression through a non-CreA related mechanism^10^.

While nearly all sugar metabolic pathways have been described in fungi^2^ the preference for sugar uptake has not been elucidated in detail. Only a recent paper described the order in which six sugars were taken up by *A*. *niger*^11^. In this study we used a broader mixture of sugars, consisting of D-glucose, D-xylose, L-arabinose, D-galactose, D-mannose, L-rhamnose, and D-galacturonic acid, all present in plant structural polysaccharides, together with D-fructose and the disaccharide maltose originating from plant storage polymers inulin and starch, respectively, as a carbon source in liquid cultures of *A*. *niger*. The uptake of the sugars from the culture liquid was followed and transcriptome analysis strongly indicated that the preferential use of the monomeric sugars is arranged at the level of transport, but it is not reflected in transcriptional regulation of sugar catabolism. In addition, we demonstrate that the main carbon catabolite repressor protein CreA does not significantly affect the order in which the sugars were taken up by *A*. *niger*.

## Results and Discussion

### *A*. *niger* consumes part of the sugars sequentially during cultivation

**Figure 1:**
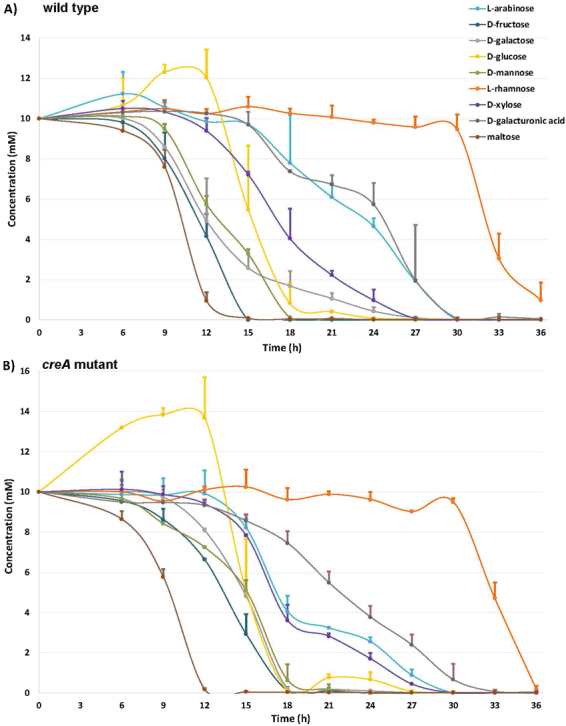
Concentration of monomeric sugars and maltose in the culture liquids of (**A**) *A*. *niger* N402 wild type and (**B**) CreA mutant. The error bars indicate the standard deviation between duplicate biological replicate cultures.

When cultivated on this mixture of sugars, *A*. *niger* imports part of them sequentially rather than simultaneously (Fig. 1A). Maltose was quickly extracellularly degraded to D-glucose, as shown by the increase of the extracellular D-glucose concentration up to 12 h. This conversion was partially masked by the rapid uptake of D-glucose. The concentration of D-fructose also quickly decreased in the culture liquids and was depleted after 15 h of incubation (Fig. 1A). D-mannose was depleted after 18 h, D-glucose after 24 h, and D-galactose and D-xylose after 27 h, although initially D-galactose was consumed faster than the three other sugar monomers. Uptake of L-arabinose and D-galacturonic acid was highly similar, starting after 15 h, and these sugars were no longer detected after 30 h. L-rhamnose was the least preferred sugar and its concentration started to decrease only after the depletion of all other sugars after 33 h, while 1 mM of extracellular L-rhamnose was still present at 36 h. These data differ from a recent study using bioreactor cultures, where D-mannose consumption did not start until D-glucose was nearly depleted and D-xylose, L-arabinose and D-galacturonic acid were consumed simultaneously^11^. As sugar transport has been shown to be pH-dependent^12,13^, this difference may be due to the ability of the fungus to modify the pH in our cultures, while the pH was kept constant (at pH 2.5) in the bioreactor study.

Considering that CreA has been suggested as the regulator mediating the use of preferred sugars first, we also analyzed sugar consumption in a *creA* deletion mutant. However, this showed that sequential sugar consumption by *A*. *niger* was not mediated by CreA, as the sugar uptake profile of the *creA* mutant was highly similar to that of the wild type (Fig. 1B). This is not due to the concentration of the sugar as we previously demonstrated that already at concentrations between 1–10 mM, CreA causes repression of several genes^14^, even though the strength of the repressing effect is dependent on the specific sugar^15^. Interestingly, in a previous study with *A*. *nidulans* in which only D-glucose and D-xylose were combined, sequential uptake was observed in the wild type, but not in a *creA* mutant^16^. However, in addition to the possible differences between *A*. *niger* and *A*. *nidulans*, this study also used bioreactors at constant pH (pH 6.0), suggesting that the difference between bioreactors and shake flasks, and possibly acidification, significantly affect sugar transport in *A*. *niger*. In the *creA* mutant maltose was converted to D-glucose slightly faster than in the cultures of the wild type strain, most probably due to high glucoamylase production in the CreA deletion strain^10^. The complete depletion of D-fructose (18 h) and D-galacturonic acid (33 h) was slightly delayed compared to the wild type.

CreA regulates the expression of several catabolic pathway genes, such as several genes involved in L-arabinose catabolism^5^, but based on our results this effect does not extend to the overall uptake of the sugars entering these pathways. This is somewhat surprising, due to several reports of individual sugar transporters being under CreA control^17–19^. Our results from the CreA mutant also demonstrate that in contrast to what was proposed in a recent study, where only the *A*. *niger* wild type was used^11^, the sequential uptake of several sugars is not mediated by CreA.

While we cannot exclude that indirect effects of CreA, e.g. by affecting acidification or the intracellular conversion of sugars, could in part hide the direct effects on sugar uptake, we think it is highly unlikely that these effects exactly compensate for the direct effects resulting is the nearly identical uptake profile we observed. No visual reduction in growth was observed for the *creA* mutant, but even if a small reduction occurred, it does not change the fact that the order of sugar uptake is identical to the wild type. In addition, the acidification in the culture of the *creA* mutant was only somewhat delayed compared to the wild type, but reached the same level (Supplementary Fig. S1), suggesting also only a small difference in growth between the strains. It is unlikely that this delayed acidification would hide an effect on sequential uptake of the sugars.

It could be that if cultures of these strains were compared in which direct spore inoculation, rather than transfers were used a larger difference would be detected, as the growth defect of the *creA* mutant (on plate) appears to be mainly during germination and early growth stages (unpublished data). However, we chose to use transfers to in fact avoid differences caused by such effects, as this allowed us to initiate the mixed sugar cultivations with the same amount of already growing mycelium. It has been demonstrated in *A*. *niger* (wild type) that not all sugars can induce germination^20^, and also not all can be transported during germination^21^, differences which have not been detected for growing mycelium. Following sugar uptake in cultures starting from spores, would therefore likely be heavily affected by the differences that occur during germination. Previous studies have also clearly demonstrated the value of transfer cultures to identify transcriptional differences between strains and conditions^8,13,14^, as this ensures the most similar possible starting point at the moment the strains are confronted with the conditions of interest. In addition, transfer cultures are also commonly used in fungal biotechnology, where strains are pre-grown at smaller scale before being transferred to the larger bioreactors. Therefore, the use of transfer cultures in our experiment also enables an easier transfer of knowledge to biotechnological companies, as it is more similar to their approach.

### Transcriptomics reveals temporal expression profiles during cultivation of *A*. *niger* on a mixture of sugars

In order to study whether the sequential use of sugars is arranged at the level of sugar uptake or catabolism, and whether this is linked to induction of CAZyme encoding genes, we performed a microarray-based expression analysis. As CreA did not appear to be regulating the uptake, gene expression was only studied in the wild type *A*. *niger* strain at four time points, 12, 18, 24 and 33 h.

Genome-wide principal component analysis (PCA) of the gene expression (Supplementary Table S1) showed the close similarity of the duplicates and the clear difference between the time points with the first time point (12 h) being most different from the others (Supplementary Fig. S2).

**Figure 2:**
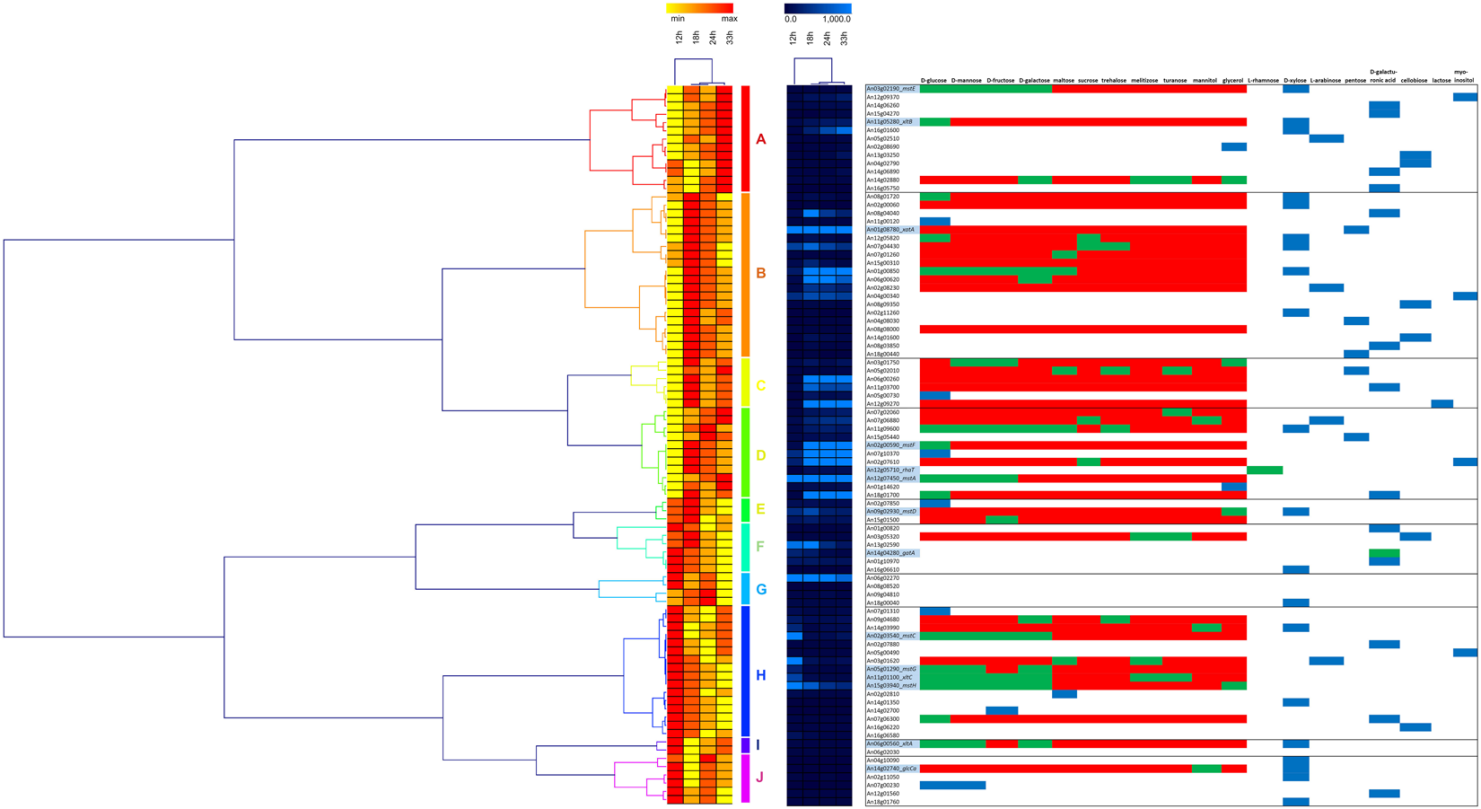
Clustering of the expression profiles of genes encoding (putative) sugar transporters. The data was normalized and expressed as linear values. The expression values were used for Hierarchical Clustering in Genesis^38^ using Pearson correlation and complete linkage and visualized using rank based (left) and traditional (right) imaging. The table on the right provides the functional and homology based annotation for the (putative) transporters. Characterized transporters in *A*. *niger* or close homologs of characterized transporters of other fungi are highlighted in pale blue in this table. Functional annotation was added based on experimental data in *A*. *niger*^1^ (green = transport of this sugar; red = no transport of this sugar). Annotation based on homology is in blue and references on which this is based can be found in Supplementary Table S2.

Little is known about sugar uptake systems in fungi, except for extensive studies in the model yeast *Saccharomyces cerevisiae*. Sugar transporters play a key role in fungal growth and adaptation to environments with different carbon sources. So far, only 42 *A*. *niger* genes have experimental evidence to support their function in sugar transport (Fig. 2, Supplementary Table S2). Distinct sets of (putative) sugar transporter encoding genes were highest expressed at the first, second and fourth time points as shown by the rank-based imaging (left cluster, Fig. 2). However, most of these genes were expressed at low level (right cluster, Fig. 2), including several of the functionally characterized transporters, suggesting that they do not play a significant role under these growth conditions. The expression patterns of some of the highly expressed sugar transporter encoding genes may explain (part of) the observed sugar uptake. However, the functional homology-based annotation should be used with caution, as it has previously been shown that sequence similarity does not always guarantee similarity in function, as transporters with different functions group together in a large scale phylogeny of *Aspergillus* putative sugar transporters^1^. In addition, it is not known whether most of these transporters are high- or low-affinity transporters, which would affect at which time point they would be most useful to the fungus.

Cluster B contains two experimentally proven hexose transporters^1^ that were mainly expressed from 18 h onwards. One of these can only transport D-galactose (An06g00620), while the other has broader specificity (An01g00850). A similar pattern was observed for a well-expressed glucose transporter from cluster D (An18g01700), coinciding with the disappearance of hexoses between 12 and 24 h. Their continued expression at the later time points may be due to traces of hexoses or a delayed shut-down mechanism of their expression. Expression of the high-affinity hexose transporter encoding gene *mstA* was high at all time points^22^, but increased expression was observed over time, which fits with the reducing D-glucose concentrations at the later time points of the cultures. A second high-affinity glucose transporter encoding gene, *mstF*^22^, was only highly expressed from 18 h onwards, while the low-affinity glucose transporter encoding *mstC*^22^ was only highly expressed at 12 h, both providing correlation between affinity and residual D-glucose concentration. Surprisingly, the latter profile was also observed for *mstG* and *mstH*, which were reported to encode high-affinity glucose transporters^23^. However, the Km of these two transporters for glucose (0.5 and 0.06 mM, respectively), was still significantly higher than that observed for MstA (0.025 mM)^13^, which may indicate that they should be considered moderate affinity rather than high-affinity transporters for *A*. *niger*.

Some other transporters without experimental proof of function were highly expressed in the cultures, suggesting a role for them in the observed uptake (Fig. 2). A putative pentose transporter of cluster B (*xatA*) was expressed during all time points, which would fit with a role in transport of both D-xylose and L-arabinose. Several putative galacturonic acid transporters were mainly expressed at the latter two time points, which coincides with the uptake of this compound. Of the three *A*. *niger* functionally validated xylose transporters, XltA, XltB and XltC^24^, slight induction was observed for *xltA* at 12 h corresponding to the start of D-xylose uptake. Expression of *xltC* was substantially induced at the first time point supporting its broad specificity for different sugars, while upregulation of *xltB* over time supported its relevance under environmental conditions with low availability of carbon sources^24^.

The D-galacturonic acid transporter encoding gene *gatA*^25^ was highly expressed at the first two time points, matching with the onset of D-galacturonic acid uptake, but downregulated after that. In contrast with the results of a previous study^25^, the regulation of D-galacturonic acid catabolic genes was not connected to the uptake of D-galacturonic acid, as these genes were in fact downregulated during D-galacturonic acid uptake. This could be due to the higher expression of the gene encoding GaaX, the repressor of the D-galacturonic acid pathway, than the gene encoding GaaR, the activator of this pathway^26^.

Overall, since most *A*. *niger* putative sugar transporters lack of biochemical functional validation of their function, we cannot link many of the genes to the sugar that is taken up, and therefore cannot fully explain the sequential uptake observed in our cultures. However, expression profiles of several known transporters match the sugar uptake profile. Therefore, the unknown transporters with distinct expression patterns in our study would be highly interesting targets for functional analysis, e.g. by expressing them in *S*. *cerevisiae* sugar transporter deletion strains.

### Temporal uptake of sugars is not reflected in temporal catabolism profiles

**Figure 3:**
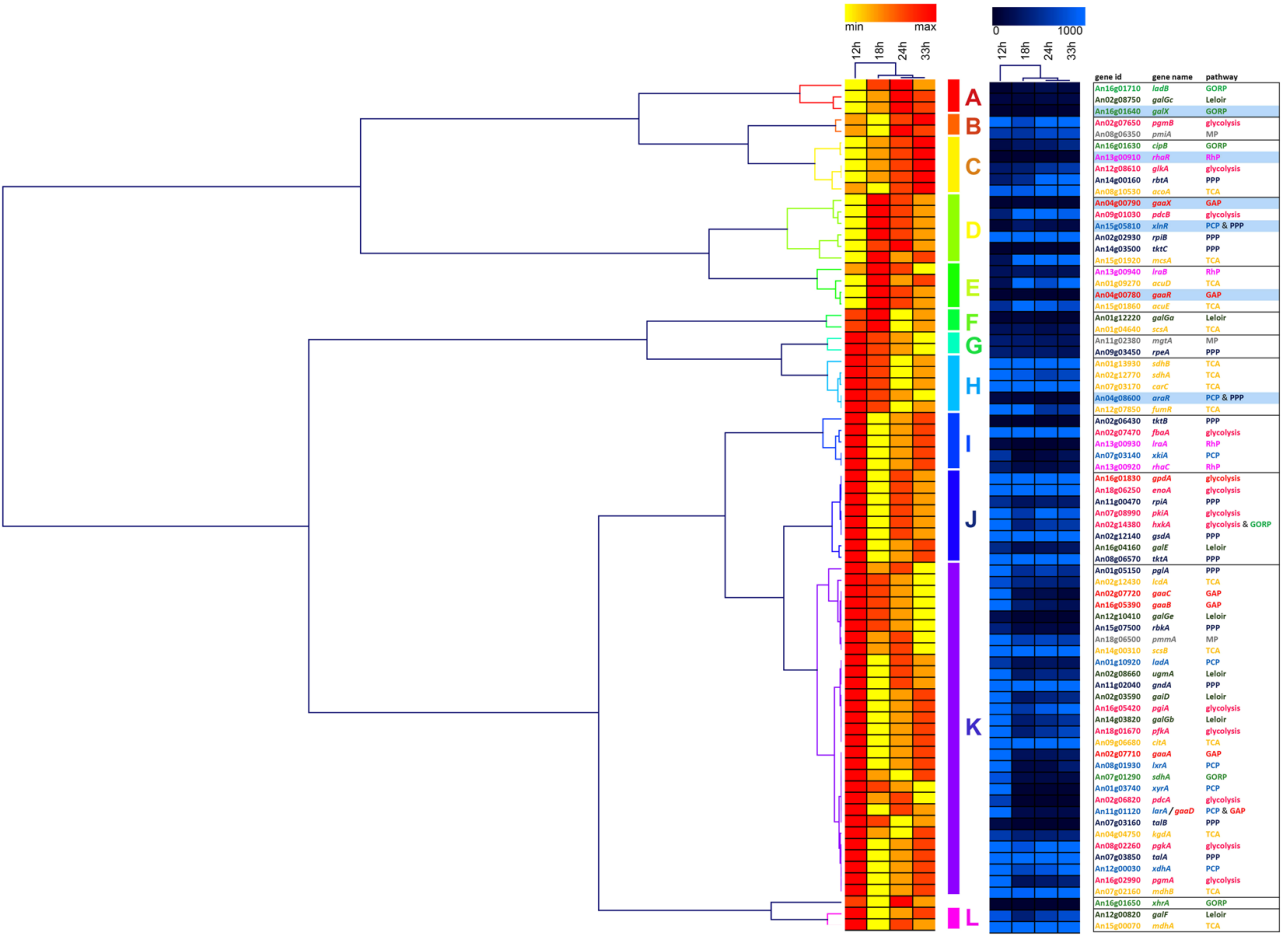
Clustering of the expression profiles of carbon metabolic genes and related regulators. The data was normalized and expressed as linear values. The expression values were used for Hierarchical Clustering in Genesis^38^ using Pearson Correlation and complete linkage and visualized using rank based (left) and traditional (right) imaging. Transcriptional regulators are highlighted in pale blue in the table on the right. Color coding of the genes in this table reflects the metabolic pathway they are related to: light blue = Pentose Catabolic Pathway (PCP), dark blue = Pentose Phosphate Pathway (PPP), red = Galacturonic Acid Pathway (GAP), light pink = Rhamnose Pathway (RhP), dark pink = glycolysis, light green = Galactose Oxido-Reductive Pathway (GORP), dark green = Leloir Pathway, grey = Mannose Pathway (MP), dark yellow = TriCarboxylic Acid pathway (TCA).

Surprisingly, the sequential uptake of sugars was not reflected in the catabolism of the individual sugar monomers, as the genes of the pathway related to a specific sugar are not specifically expressed when that sugar is taken up. E.g. several genes of the D-galactose catabolic pathways increase in expression from 18 to 33 h, while galactose uptake occurs mainly in the first 12–18 h. In contrast, D-galacturonic acid uptake occurs after 18 h, while the highest expression of this pathway is at 12 h (Supplementary Fig. S2). Therefore, the physiology of *A*. *niger* on the mixture of (monomeric) sugars is very different from that detected on individual sugars in liquid cultures^27^. The cluster analysis of the sugar catabolic gene expression data showed that the earliest time point (12 h) differed most from the three later time points (Fig. 3, Supplementary Table S3). Except for a general up-regulation of many of the catabolic genes at 12 h, there was no specific temporal expression profile for the genes representing any complete sugar catabolic pathway, as indicated in more detail by the individual pathway figures (Supplementary Fig. S3). For instance, only three genes of the pentose catabolic pathway show an increasing profile from 18 to 33 h (Supplementary Fig. S3D), and the same is observed for part of the Leloir pathway genes (Supplementary Fig. S3B). Similarly, only two of the three L-rhamnose catabolic genes have a strong increase in expression from 24 to 33 h (Supplementary Fig. S3C). The genes from the TCA cycle and glyoxylate shunt were highly expressed throughout the studied time points, indicating that *A*. *niger* was not under carbon starvation in the cultures (Supplementary Fig. S3). Also glycolytic genes were expressed in all time points, but the majority of them showed the highest expression at the earliest time point (12 h), which coincides with uptake of the hexoses, which enter glycolysis.

Surprisingly, the genes from the L-rhamnose pathway were upregulated at the early time points, which does not match the L-rhamnose uptake profile. Also, the expression of the gene encoding the L-rhamnose pathway regulator, RhaR^28^, was slightly induced over time, but did not show substantial upregulation at 33 h when L-rhamnose was taken up. This may be due to the uptake of micromolar amounts of L-rhamnose, not detectable with the HPAEC analysis that could be sufficient for induction of the genes. The recently identified L-rhamnose transporter^29^ is expressed at a constant level from 18 h onwards (Supplementary Table S2), which also does not correlate with the disappearance of L-rhamnose from the medium.

### CAZy genes under control of the same regulator do not have similar temporal expression profiles

**Figure 4:**
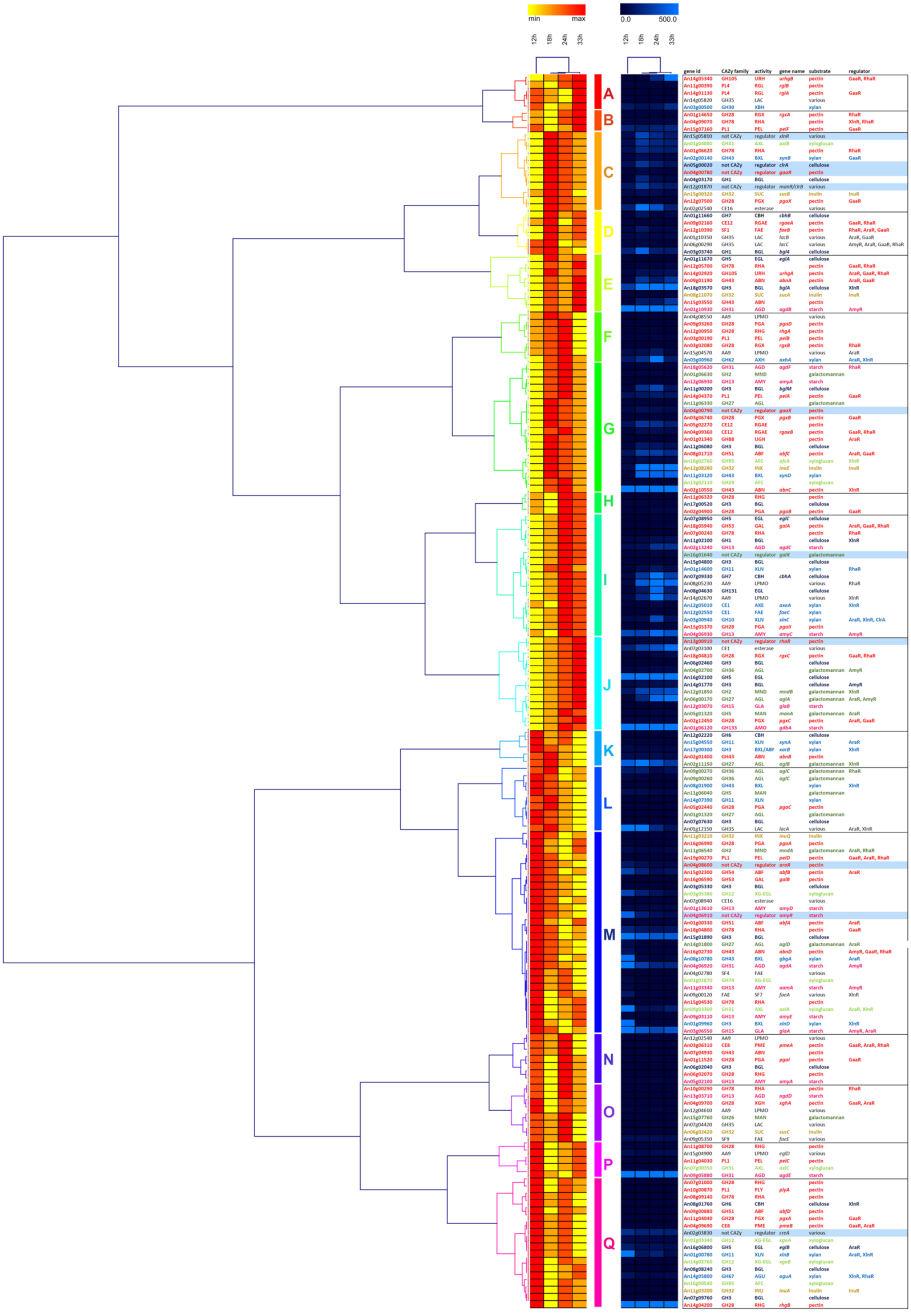
Clustering of the expression profiles of genes encoding plant biomass active CAZymes and related regulators. The data was normalized and expressed as linear values. The expression values were used for Hierarchical Clustering in Genesis^38^ using Pearson correlation and complete linkage and visualized using rank based (left) and traditional (right) imaging. Transcriptional regulators are highlighted in pale blue in the table on the right. Control of these regulators on CAZy genes^26^ has been indicated in the last column of this table. Color coding of the genes in this table reflects the substrate they are related to: black = various substrates, pale green = xyloglucan, dark green = galactomannan, pink = starch, light blue = xylan, dark blue = cellulose, red = pectin, orange = inulin.

The cluster analysis of the CAZy gene expression data showed no obvious patterns in terms of target substrates or function of the corresponding enzymes (Fig. 4), which indicates that the genes encoding (extracellular) polysaccharide degrading enzymes are induced by different compounds. In addition, the *A*. *niger* CAZy genes with temporal expression profiles are not controlled by a common regulator based on recent study^27^ (Fig. 4). For example, the large cluster G contains genes related to the degradation of nearly all plant biomass polysaccharides. Also, genes that have been shown to be controlled by the same transcriptional activator^27^, do not cluster in our data (Fig. 4), indicating a much more complex regulatory system when *A*. *niger* is exposed to a mixture of monosaccharides.

In conclusion, the results of our study demonstrate a highly selective and sequential uptake of different sugars by *A*. *niger* that is not mediated by the major carbon catabolite repressor protein CreA. The absence of correlation of sugar uptake and expression of the genes of the related catabolic pathways in our study suggest that sugar transport and metabolism are highly separated physiological processes when *A*. *niger* grows in liquid cultures.

## Methods

### Fungal strains and cultivation

*A*. *niger* N402 wild type^30^ and CreA mutant^31^ were pre-cultured in 1 L Erlenmeyer flasks in 200 mL complete medium (CM), pH 6.0, that contained *Aspergillus* minimal medium (MM)^32^. The pre-cultures were inoculated with 10^6^ spores/mL and incubated at 250 rpm for 16 h at 30 °C. Mixture of 10 mM monomeric sugars (D-glucose, D-fructose, D-mannose, D-galactose, L-rhamnose, D-xylose, L-arabinose, D-galacturonic acid) and maltose (Sigma-Aldrich) was added into 50 mL MM in 250 mL Erlenmeyer flasks. This concentration was used as previous studies demonstrated that at concentrations below 10 mM, growth reduction occurs (R.P. de Vries, unpublished data). The cultures were inoculated with mycelial aliquots from the pre-cultures (1 g wet-weight per flask) and incubated at 250 rpm, at 30 °C. The duplicate cultures were sampled every three hours from 6 h up to 36 h by collecting 1 mL of culture liquid. The samples were centrifuged and stored at −20 °C prior to analysis of residual sugar concentration.

### Determination of sugars

The levels of monomeric sugars and maltose in the culture liquid supernatants were determined by using high performance anion-exchange chromatography (HPAEC, Thermo) as previously described^33^.

### Microarray processing and analysis

Samples from 12, 18, 24 and 33 h were used for microarrays to evaluate the influence of the changes in extracellular sugar concentration on whole genome gene expression. These time points were chosen as they reflect the stage in which specific sugars were taken up. RNA was extracted using TRIzol reagent (Invitrogen) and purified using TRIzol® Plus RNA Purification Kit (Sigma-Aldrich) according to the instructions of the manufacturer. The RNA concentration was calculated from the absorbance at 260 nm in a spectrophotometer (Biochrom Libra S22). The RNA quality was analyzed with an Agilent 2100 bioanalyzer using a RNA6000 LabChip kit (Agilent Technology). Microarray hybridization was performed at GenomeScan (Leiden, The Netherlands). Microarray data was analyzed using Affymetrix Expression Console software v1.4.1.46 with the normalized values in linear scale. The probe intensities were normalized for background by the robust multiarray average (RMA) method which makes use of only the perfect match probes. Gene expression values were calculated with the medianpolish summary method from the PM probes. Genome scale PCA analysis with gene expression of the four different time points was generated using FactoMineR^34^ package from Rcomander v.2.1–7 in R 3.1.2.^35^.

### Discovery of sugar transporters

A total of 71 known fungal transporter sequences were collected from Transporter classification database^36^ (TCDB, http://www.tcdb.org/) and by manual literature search. The sugar transporter domain (PF00083) profile extracted from PFAM database (http://pfam.xfam.org/family/pf00083) was used to search against the combined sequence files of *A*. *niger* proteome and known transporters with the ‘hmmsearch’ of the HMMER tool^37^. The hmmsearch score ≥238 was chosen as a cutoff to define the sugar transporter candidates since it was the lowest score observed among the search results of all the known transporters. Each transporter candidate was Blast searched against the collected sequences of known fungal transporters. With the threshold of Blast e-value < 1E-6 and identity percent >30%, the best Blast hit was used to annotate the candidate transporters.

The raw and normalized microarray data generated and analysed during the current study are available in the Gene Expression Omnibus (GEO, NCBI) database under the accession number GSE98434.

## Electronic supplementary material

Supplementary Dataset 1

Supplementary Dataset 2
