# Supplementary material for: The fungus Aspergillus niger consumes sugars in a sequential manner that is not mediated by the carbon catabolite repressor CreA
Source: Sci Rep. 2018 Apr 27;8:6655. doi: 10.1038/s41598-018-25152-x (PMC5923239; doi:10.1038/s41598-018-25152-x)

The fungus *Aspergillus niger* consumes sugars in a sequential manner that is not mediated by the carbon catabolite repressor CreA

Miia R. Mäkelä[1,2], María Victoria Aguilar-Pontes[1], Diana van Rossen-Uffink[1], Mao Peng[1], Ronald P. de Vries[1,2]*

[1]Fungal Physiology, Westerdijk Fungal Biodiversity Institute & Fungal Molecular Physiology, Utrecht University, Utrecht, the Netherlands

[2]Division of Microbiology and Biotechnology, Department of Food and Environmental Sciences, University of Helsinki, Helsinki, Finland

Supplementary Data 1: Combines Supplementary Figure 1, 2 and 3

Supplementary Figure S1. pH profile of the wild type and CreA cultures.

Mycelium was pre-cultured in complete medium with 2% D-fructose for 18 h and then transferred to minimal medium with the mixture of sugars. Starting pH after transfer was pH 6.0. Cultures were performed in triplicate and averages and standard deviations are plotted in the graph.

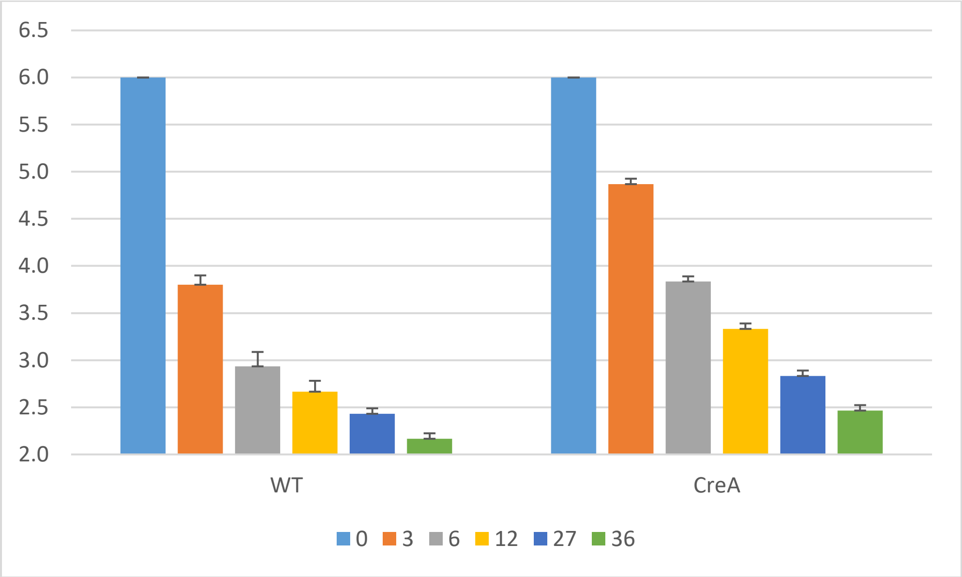

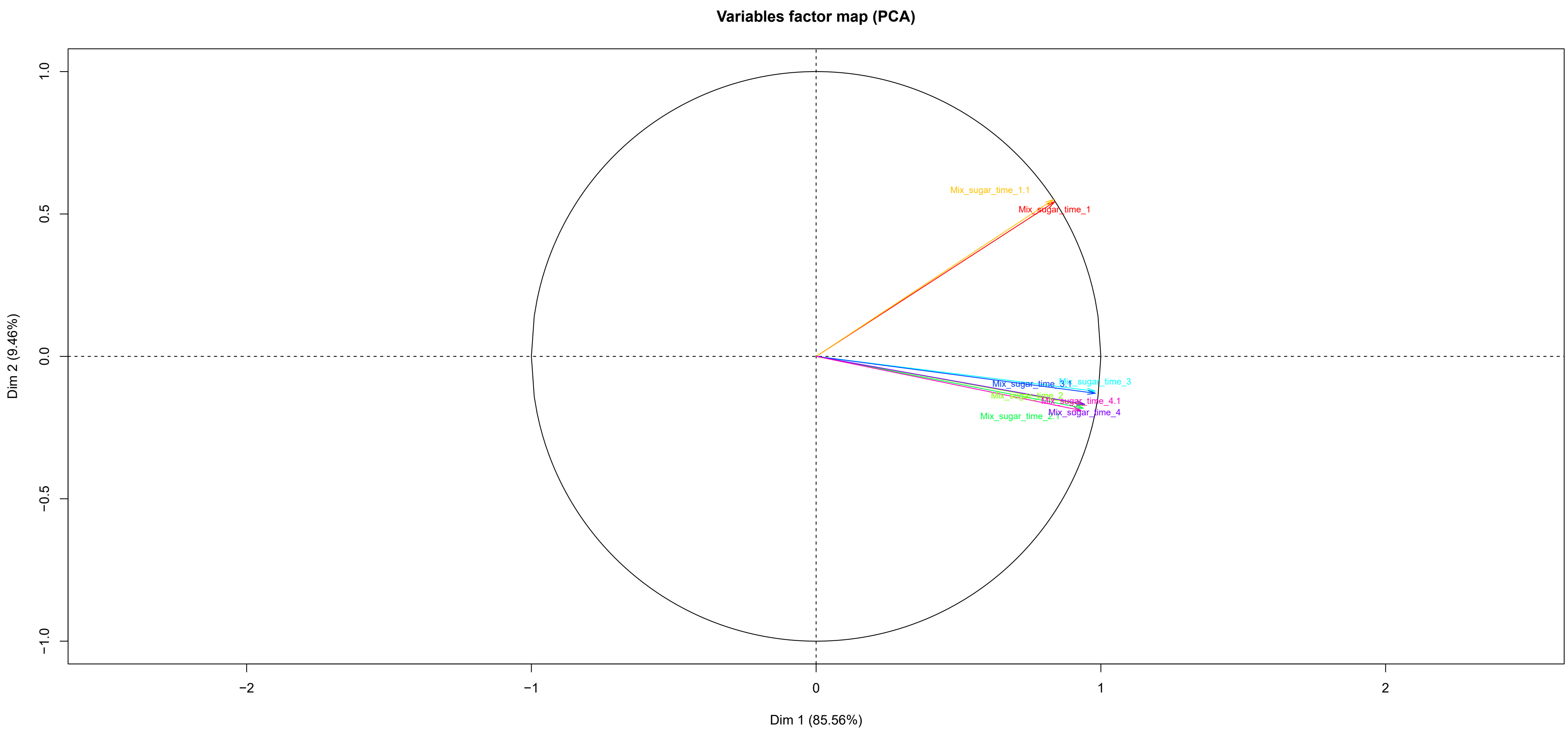

Supplementary Figure S2. Principal component analysis (PCA) of the microarray data. The graph shows the close similarity between the replicates as well as the significant difference the first time point has from the latter three timepoints.

Supplementary Figure S3. Representation of sugar catabolic pathways, including expression profiles of the genes involved in the pathways. A) Glycolysis, mannose catabolism and TCA cycle. Values at the Y-axis are signal values of the genes.

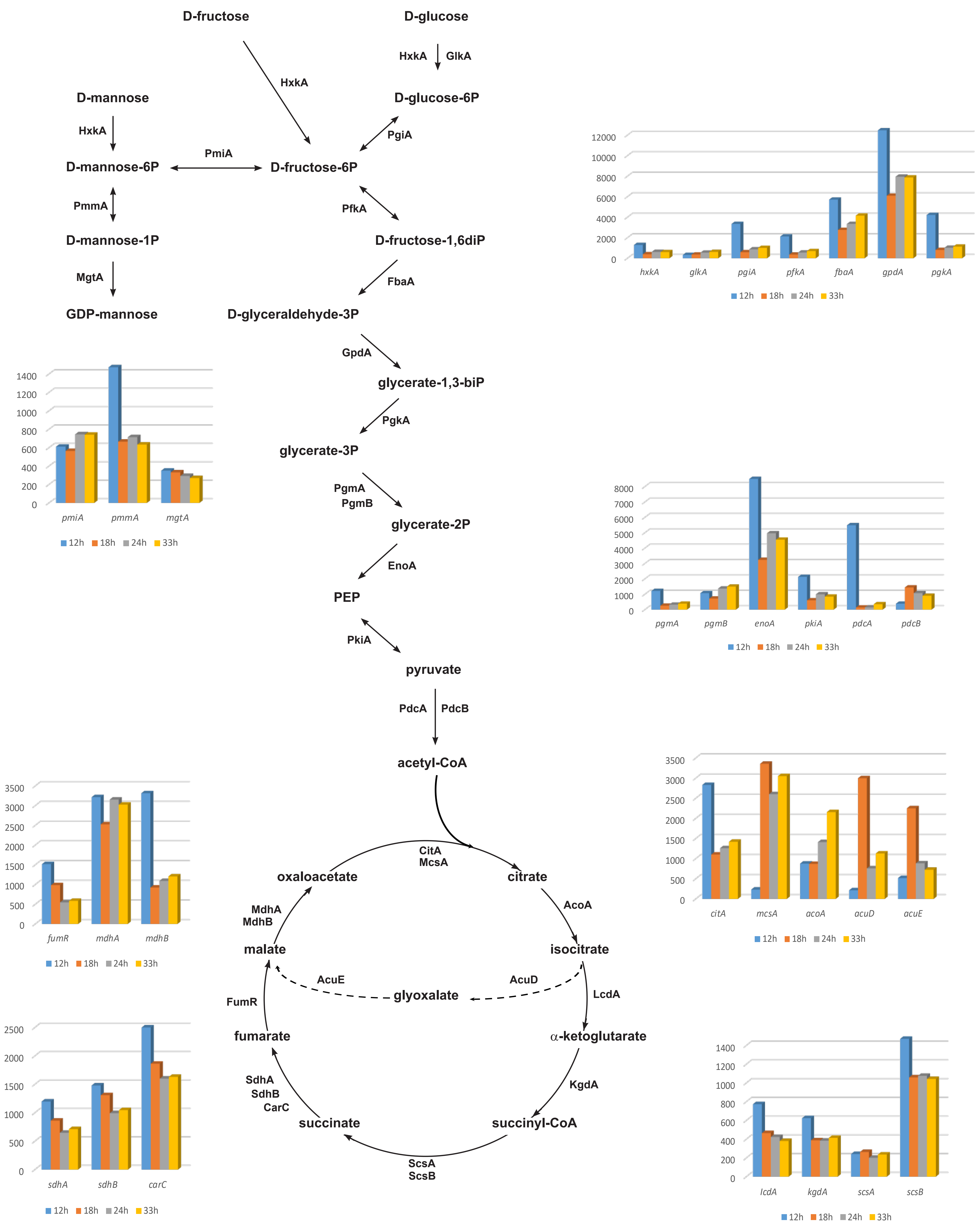

Supplementary Figure S3. B) Oxido-reductive (left) and Leloir (right) D-galactose pathway. The galactose-responsive transcriptional activator encoding gene *galX* is also included. Values at the Y-axis are signal values of the genes.

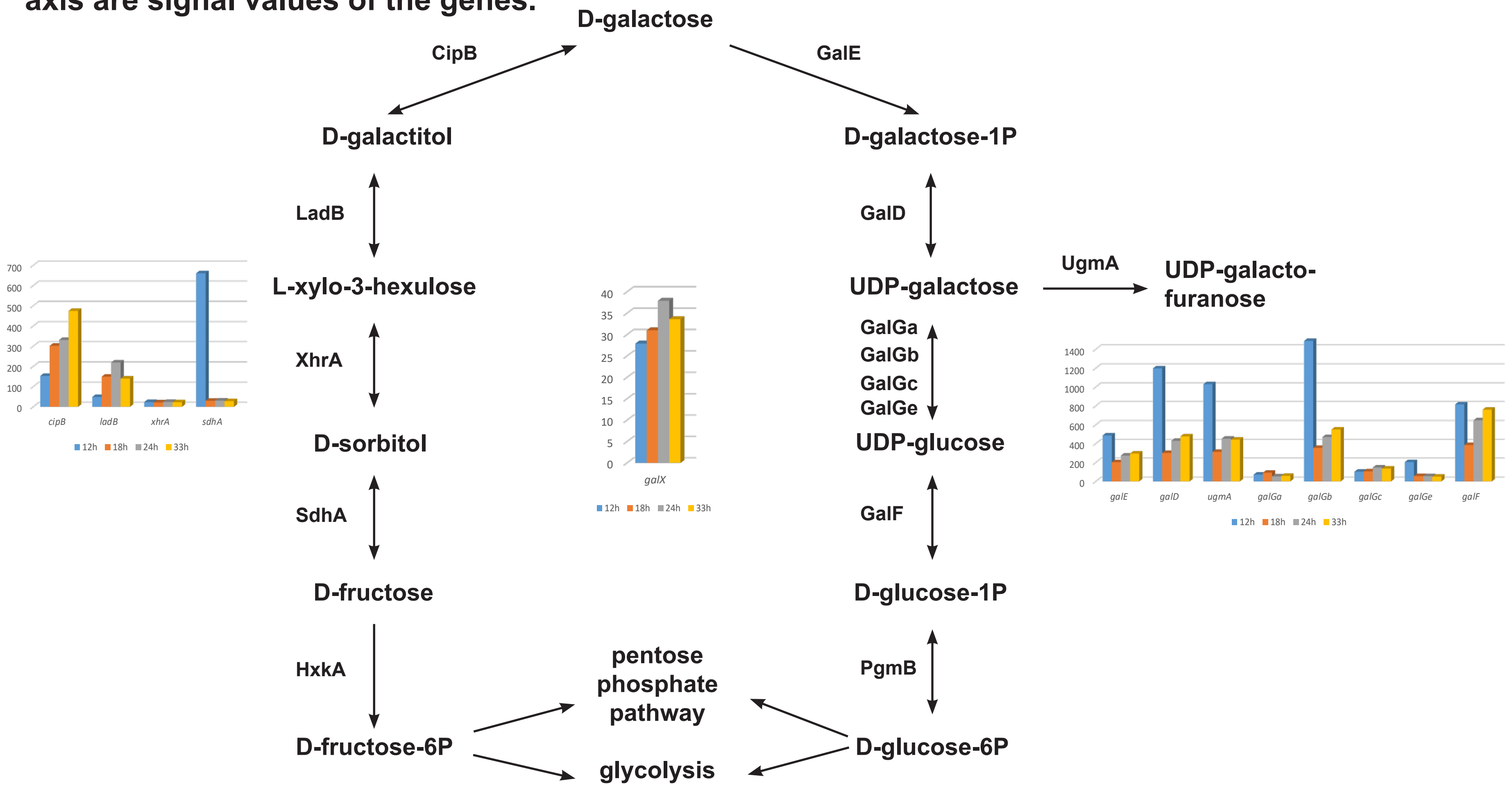

Supplementary Figure S3. C) L-rhamnose and D-galacturonic acid catabolic pathways. The genes encoding the pathway specific regulators (*rhaR*, *gaaR* and *gaaX*) are also indicated. Values at the Y-axis are signal values of the genes.

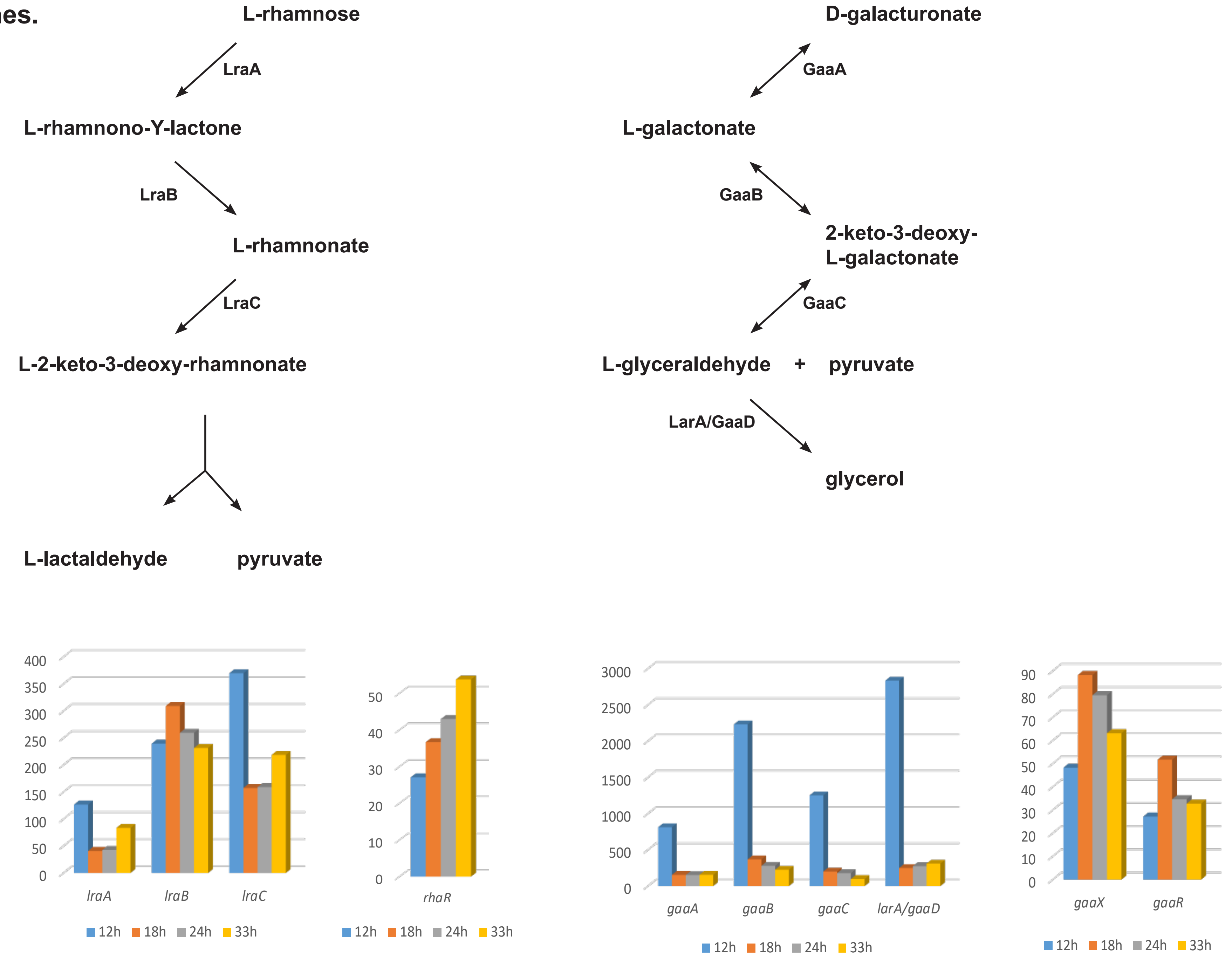

Supplementary Figure S3. D) Pentose catabolic and pentose phosphate pathway. The genes encoding the pentose responsive transcriptional activators (*araR* and *xlnR*) are also indicated. Values at the Y-axis are signal values of the genes.

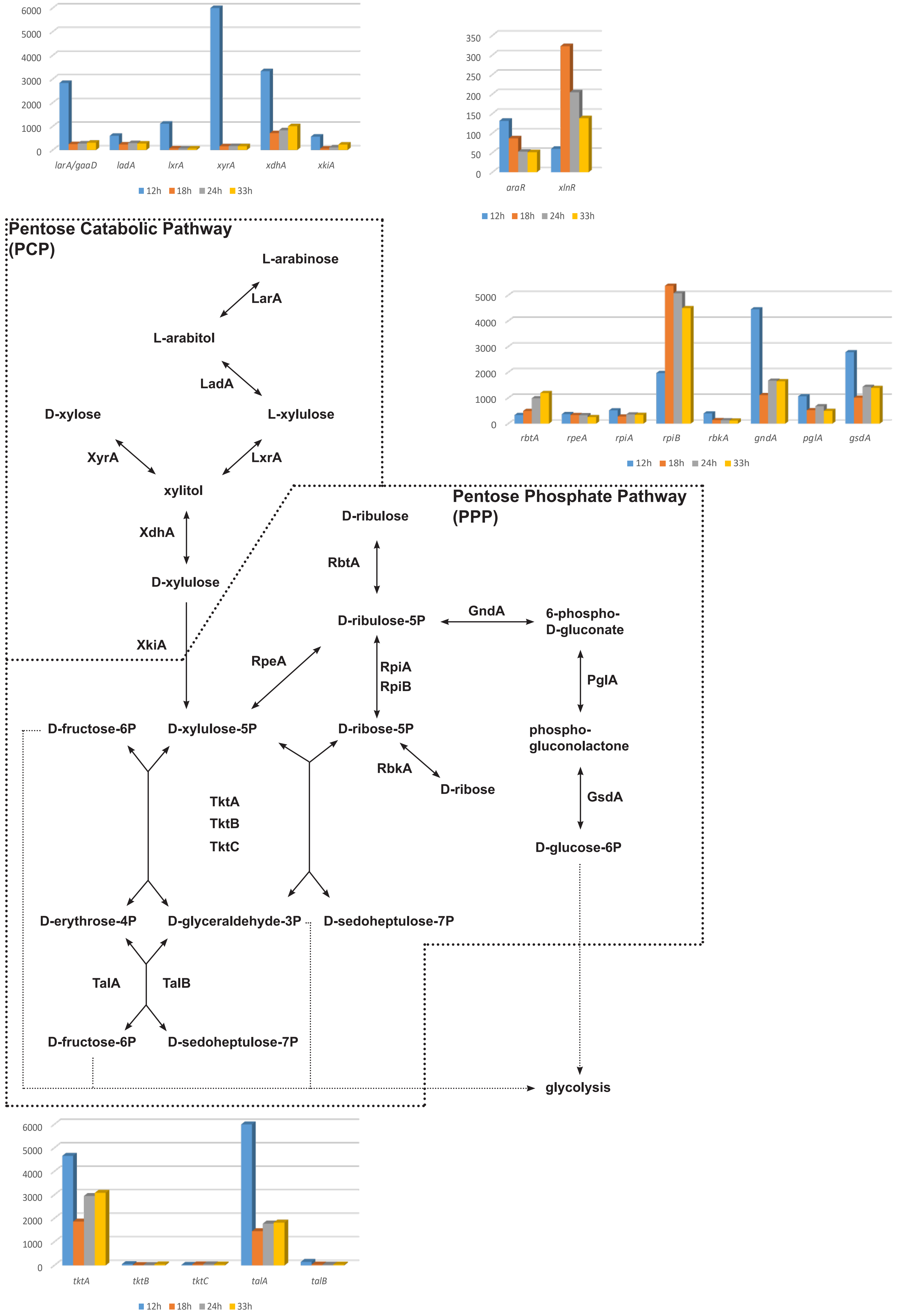

Supplement: Supplementary file 1 — Supplementary Dataset 1 [file 41598_2018_25152_MOESM1_ESM.pdf]
